# Effect of cyclosporine A on focal segmental glomerulosclerosis caused by MYO1E mutation in a Chinese adult patient: A case report

**DOI:** 10.1097/MD.0000000000032683

**Published:** 2023-01-27

**Authors:** Ruizhao Li, Wei Dong, Yingwen Chen, Tianwei Tang, Xingchen Zhao, Li Zhang, Xinling Liang

**Affiliations:** a Department of Nephrology, Guangdong Provincial People's Hospital (Guangdong Academy of Medical Sciences), Southern Medical University, Guangzhou, Guangdong, China; b Guangdong Cardiovascular Institute, Guangdong Provincial People's Hospital, Guangdong Academy of Medical Sciences, Guangzhou, Guangdong, China; c School of Medicine, South China University of Technology, Guangzhou, China.

**Keywords:** focal segmental glomerulosclerosis, myosin 1e, cyclosporine A

## Abstract

Focal segmental glomerulosclerosis (FSGS) describes a renal histologic lesion with diverse causes and pathogenicities. Monogenic abnormalities which are associated with impaired function of podocyte could result in FSGS. Most of genetic FSGS do not respond to immunosuppressive agents and often develop end-stage kidney disease. We reported a case of FSGS caused by myosin1e (MYO1E) mutation, alleviated by cyclosporine A (CsA) and low-dose glucocorticoid.

**Patient concerns:**

The patient was a 38-year-old male with nephrotic range proteinuria. He didn’t respond to prednisone 65mg/day. Kidney biopsy in our hospital showed FSGS with several hypoplasia and tiny loops. In addition, focal thickening and disorganization of the glomerular gasement membrane as well as diffuse foot process effacement were observed in electron microscope.

**Diagnoses:**

Genetic testing indicated homozygous deletion mutation of MYO1E. The patient was diagnosed with genetic FSGS caused by MYO1E homozygous mutation.

**Interventions:**

The patient was treated with CsA 50mg twice a day and low-dose methylprednisolone.

**Outcomes:**

CsA and low-dose glucocorticoid dramatically reduced proteinuria, and partial remission was attained in 3 years follow-up.

**Lessons:**

MYO1E autosomal recessive mutation was a rare FSGS causative mutation that might benefit from CsA treatment. However, the long-term effect of CsA on FSGS caused by this mutation should be investigated in the future.

## 1. Introduction

Focal segmental glomerulosclerosis (FSGS) is a clinicopathologic entity characterized by massive proteinuria and podocyte injury, always with nephrotic syndrome. FSGS refers to a segmental increase in glomerular matrix with obliteration of the capillary lumina in at least 1 glomerulus, including collapsing, tip lesion, cellular, perihilar variant, and FSGS not otherwise specified variant.^[1]^ FSGS is not a single disease but covers a diverse group of diseases with diverse etiologies. The traditional pathological classification has no indication for etiology and treatment approaches in patients with FSGS lesions. KDIGO proposed changes to the nomenclature of FSGS to improve clinical utility and provide clarity about the underlying pathophysiology which encompasses primary, genetic, secondary and undetermined causes.^[2]^ Primary FSGS always respond to immunosuppression, while treatment resistance is a common feature in genetic forms of FSGS.^[3]^ Effective and specific therapies on genetic FSGS are still lacking, and patients with genetic FSGS often progress to end-stage kidney disease.^[4]^ In this article, we reported a Chinese FSGS patient with myosin 1E (MYO1E) mutation for whom cyclosporine A (CsA) was effective.

## 2. Case report

The patient was a 38-year-old male with nephrotic range proteinuria. 1 year before admission to our hospital, proteinuria was found in health screening. His urine dipstick showed 3 + for protein and 1 + for blood. Immediately, further evaluation showed urinary protein excretion is 4.5g/gCr and serum creatinine is 87μmol/L (eGFR determined by CKD-EPI equation was 97.61mL/minute/1.73m^2^). Then corticosteroid therapy (prednisone 65mg/day) was initiate in another hospital without renal biopsy. After 2 months of corticosteroid therapy, proteinuria was not relieved, and additional CsA (75mg twice a day) treatment was administered. This patient self-reported that proteinuria could be relieved partially after CsA treatment without the original urine test record. 1 month ago, this patient discontinued prednisone and CsA on his own. This patient didn’t have any symptom. He had gout for 4 years without regular treatments and didn’t take any non-steroidal anti-inflammatory drugs. There was no known family history of kidney disease. However, his parents are married parallel cousins. The blood pressure was 140/97 mm Hg. The remainder of the physical examination was normal. Initial laboratory tests at our hospital showed nephrotic range proteinuria (urinary protein excretion, 3.62g/gCr; 24 hours urine protein quantification, 5.32g), hypoalbuminemia (albumin [ALB] 32.6 g/L) and mildly elevated serum creatinine (serum creatinine 117.27*μ*mol/L; eGFR determined by CKD-EPI equation, 67.7mL/minute/1.73m^2^). Autoantibodies (ANA, ENA, anti-DNA, ANCA, anti-PLA2R) were undetectable, and serum C3 and C4 complement components were normal. A screening test for human immunodeficiency virus, hepatitis B virus and hepatitis C virus were negative. Ultrasound revealed normal-sized kidneys with corticomedullary demarcation. No signs of tumors were detected by abdominal ultrasonography and thorax CT. Thrombosis was not observed on color doppler ultrasonography of lower extremity veins and renal vein. The other laboratory test results are shown in Table 1.

**Table 1:** Laboratory data at our hospital.

Variable	Reference range	Value in this patient
Blood routine		
White blood cell (×10^9^/L)	3.5–9.5	10.64
Hemoglobin (g/L)	115–150	151
Platelets (×10^9^/L)	125–135	169
Urine routine		
Protein	Negative	3+
Red blood cell	Negative	1+
Urine protein to creatinine ratio (g/gCr)	<50	3.62
24 h urine protein quantification (g)	<0.1	5.32
24 h urine albumin quantification(g)	<0.03	3.7
Serum creatinine (μmol/L)	57–111	117.27
Blood urea nitrogen (mmol/L)	3.1–8.8	6.44
Total protein (g/L)	65–85	55.4
Albumin (g/L)	40–55	32.6
Cholesterol (mmol/L)	3.8–6.22	4.90
Triglyceride (mmol/L)	0.4–1.53	1.45
Fasting glucose (mmol/L)	3.90–6.10	4.22
Uric acid (μmol/L)	155–357	656.5
D-dimer (ng/mL)	<500	240
C3	1289	900–1800
C4	332	100–400

**Figure 1.**
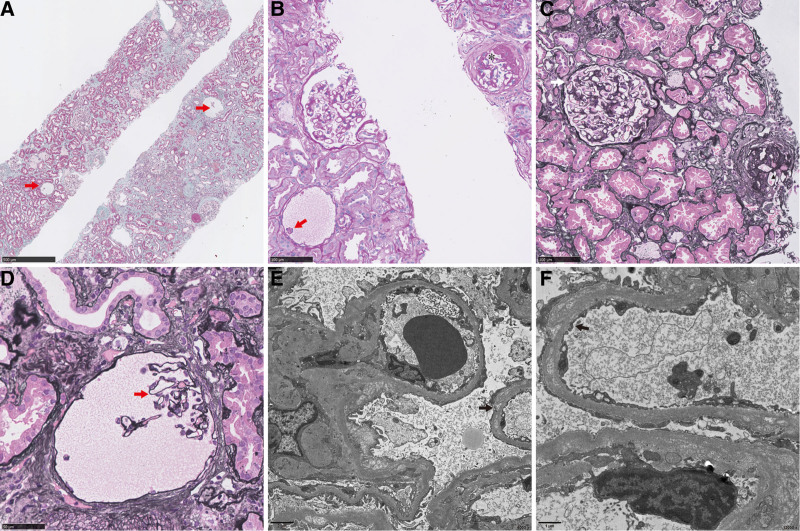
Renal pathology of this patient. (A). Masson stain, Periodic acid-Schiff stain, original magnification ×50. (B). Periodic acid-Schiff stain, original magnification ×200. (C). Periodic acid-silver methenamine stain, original magnification ×200. (D). Periodic acid-silver methenamine stain, original magnification ×400. (E). Electron micrograph, original magnification ×6000. (F). Electron micrograph, original magnification ×12,000. Red arrow, hypoplasia and tiny glomerular capillary loops. Asterisk, segmental sclerosis. Triangle, glomerular sclerosis. White arrow, tuft adhesion to the bowman capsule. Black arrow, focal thickening and disorganization of the GBM. GBM = glomerular gasement membrane.

Kidney biopsy was performed. Pathological examination of light microscopy (shown in Fig. 1A–D) showed 8 out of 26 glomeruli were glomerular sclerosis and 1 glomerulus was segmental sclerosis. Tuft adhesion to the bowman capsule were found in 3 glomeruli. Different from glomerular lesions of other common FSGS, there were several hypoplasia and tiny loops. The range of tubular atrophy and interstitial fibrosis were about 40% with tubular vacuolar degeneration and interstitial foam cells. Immunofluorescent staining was negative for IgA, IgG, IgM, C3, C4, and C1q staining. In addition to diffuse foot process effacement, electron microscope also revealed focal thickening and disorganization of the glomerular gasement membrane and loss of identifiable layers (shown in Fig. 1E, F). Because this patient had glucocorticoid resistance, unusual pathological findings, and family with consanguineous marriage, genetic FSGS was suspected. Thus, whole exome sequencing was performed and valsartan 80 mg was administrated without immunosuppressive therapy.

1 month later, whole exome sequencing result indicated suspected homozygous deletion mutation about 0.216 kb in chr15q22.2 including whole exon 10 of MYO1E. Sanger sequencing confirmed the homozygous deletion of whole exon 10. Sanger sequencing was also detected in his 2 sons who had no proteinuria, and revealed heterozygous deletion mutation. Finally, genetic FSGS caused by MYO1E homozygous mutation was diagnosed.

**Figure 2.**
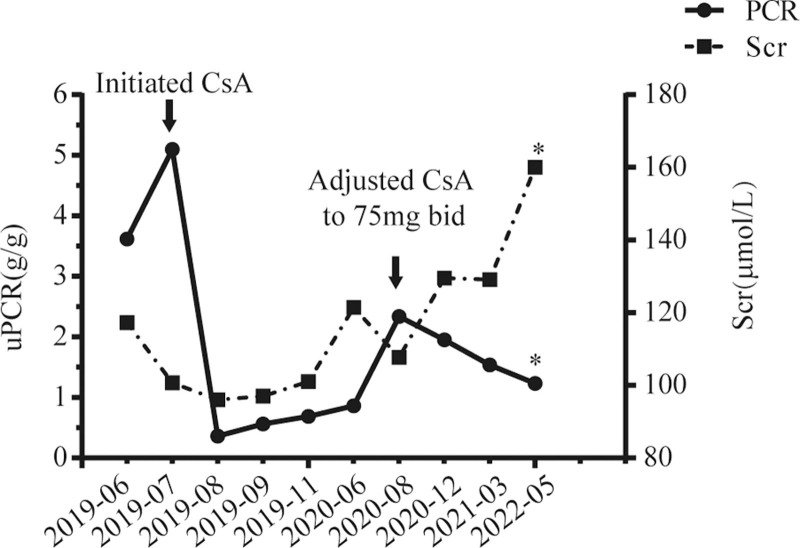
Follow up of this patient. *, laboratory data in local hospital. uPCR = urine protein to creatinine ratio, Scr = serum creatinine, CsA = cyclosporine A.

Proteinuria was not relieved after valsartan treatment and serum ALB decreased to 29.0g/L. Previous report found CsA might be effective against FSGS caused by MYO1E mutation to a certain extent.^[5]^ We treated the patient with CsA 50mg twice a day and low-dose methylprednisolone (8 mg/day). It is striking that urinary protein excretion was reduced to 0.36 g/gCr and serum ALB recovered to 35.0g/L just after 1 month treatment. Though proteinuria rebounded transiently because of inadequate serum CsA concentration in subsequent treatment, proteinuria decreased to 1.23 g/gCr after increased CsA dose to 75mg twice a day (shown in Fig. 2). At the end of 3 years follow up, serum creatinine increased mildly to 160.00*μ*mol/L and no obvious side effects of CsA was observed.

## 3. Discussion and Conclusion

Gene mutation is an important etiology of FSGS. Even in adult FSGS patients, the genetic diagnostic rate was 11%.^[6]^ Most of the protein products of these monogenic FSGS genes localize to the podocyte which constitute the outer epithelial layer of the 3-layered capillaries in the glomerular filter.^[7]^

MYO1E autosomal recessive mutation was reported as a rare FSGS causative mutation, accounting for 0.95% to 2% of monogenic causes of FSGS.^[8,9]^ Myo1e is a membrane-associated class I myosin with a motor-head domain (includes a P-loop, a Switch-1 and a Switch-2 domains) that binds ATP and F-actin, a light-chain binding neck domain (IQ) that binds calmodulin and a tail, containing lipid binding tail homology-1, proline-rich tail homology-2 and Srchomology-3 domains.^[10–12]^ Although Myo1e is expressed in several tissues, no study had report patients with MYO1E mutation or Myo1e-deficient mice have any extrarenal involvement.^[5,13,14]^ Myo1e specifically localizes to podocytes in the kidney as a key component of foot process cytoskeleton and necessary for normal glomerular filtration.^[5,13,14]^ MYO1E mutation or knockout would lead to impaired podocyte migration and foot process effacement, exhibiting signs of FSGS.^[5,13,14]^ Although MYO1E mutation in Chinese children with steroid-resistant nephrotic syndrome had been reported in a study, kidney biopsy was not performed to confirm the pathological changes.^[15]^ We reported the first Chinese adult genetic FSGS caused by MYO1E mutation which confirmed by pathological and genetic examination. Pathological finding of this case was almost consistent with the previous reported cases which exhibited FSGS with thickened and disorganized glomerular basement membrane.^[5]^ Additionally, in this case we found several hypoplasia and tiny loops which were not reported in other studies about MYO1E mutation. We speculated the pathological differences between this case and the previous cases might resulted from distinct mutation. All the previous reported MYO1E mutation were point mutation (missense or nonsense mutation).^[5,16]^ The reported missense mutation replaced alanine-159 of Myo1e by proline which located in the Switch-1 region and affected binding ATP and F-actin. The reported nonsense mutation causes protein interruption at tyrosine 695 (p.Y695X), at the start of the calmodulin-binding domain. While we firstly reported large fragment deletion mutation of MYO1E which leaded loss of corresponding protein residues in the exon 10 (aa 304–309). The deleted amino acid located in motor-head domain close to Switch-2 domains, and might also affected binding ATP and F-actin but in a different manner.

FSGS patients with genetic alterations almost impossibly response to immunosuppressive agents and often develop end-stage kidney disease necessitating dialysis and/or transplantation.^[4]^ In previous study, 4 FSGS patients with MYO1E mutation were treated with CsA, and 3 patients achieved partial remission.^[5]^ We also treated this patient with CsA and low-dose glucocorticoid and followed up for 3 years. During the initial phase of treatment, proteinuria was miraculously reduced. Although proteinuria rebounded in the further flow-up, partial remission was attained. MYO1E mutation would affect cytoskeletal abnormalities and function of podocyte, resulting impaired glomerular filtration barrier. Cyclosporine could not only inhibit T cell function but also stabilize the actin cytoskeleton of the podocyte by blocking calcineurin.^[17–19]^ Therefore, cyclosporine might help improve the cytoskeletal abnormalities that are induced by the abnormal MYO1E protein.

In our report, we have presented first Chinese adult genetic FSGS caused by MYO1E deletion mutation. We treated this patient with CsA and low-dose glucocorticoid. This patient was followed up for 3 years and achieve partial remission. However, the long-term effect of CsA on FSGS caused by MYO1E mutation should be investigated in the future.

## Author contributions

**Conceptualization:** Xinling Liang, Ruizhao Li.

**Data curation:** Wei Dong, Yingwen Chen, Tianwei Tang, Xingchen Zhao, Li Zhang.

**Resources:** Ruizhao Li, Wei Dong.

**Writing – original draft:** Wei Dong, Ruizhao Li.

**Writing – review & editing:** Xinling Liang.
